# Do Abiotic Stresses Affect the Aroma of Damask Roses?

**DOI:** 10.3390/plants12193428

**Published:** 2023-09-28

**Authors:** Nutthawut Charoimek, Sirinun Phusuwan, Chaleerak Petcharak, Kiattisak Huanhong, Shashanka K. Prasad, Taepin Junmahasathien, Julaluk Khemacheewakul, Sarana Rose Sommano, Piyachat Sunanta

**Affiliations:** 1 Department of Pharmaceutical Science, Faculty of Pharmacy, Chiang Mai University, Chiang Mai 50200, Thailand; nutthawut_charoimek@cmu.ac.th (N.C.); phoenixj035@hotmail.com (T.J.); 2 Plant Bioactive Compound Laboratory (BAC), Faculty of Agriculture, Chiang Mai University, Chiang Mai 50200, Thailand; kiattisak_huanhong@cmu.ac.th (K.H.); shashankaprasad@jssuni.edu.in (S.K.P.); sarana.s@cmu.ac.th (S.R.S.); 3 Department of Plant and Soil Sciences, Faculty of Agriculture, Chiang Mai University, Chiang Mai 50200, Thailand; sirinun_p@cmu.ac.th (S.P.); chaleerak_petch@cmu.ac.th (C.P.); 4 Department of Animal and Aquatic Science, Faculty of Agriculture, Chiang Mai University, Chiang Mai 50200, Thailand; 5 Department of Biotechnology and Bioinformatics, School of Life Sciences, JSS Academy of Higher Education and Research, Mysuru 570015, Karnataka, India; 6 Center of Excellence in Agro Bio-Circular-Green Industry (Agro BCG), Faculty of Agro-Industry, Chiang Mai University, Chiang Mai 50100, Thailand; julaluk.kh@cmu.ac.th; 7 Division of Food Science and Technology, Faculty of Agro-Industry, Chiang Mai University, Chiang Mai 50100, Thailand; 8 Multidisciplinary Research Institute, Chiang Mai University, Chiang Mai 50200, Thailand

**Keywords:** damask rose, secondary metabolite, VOCs, drought, chemical composition

## Abstract

Roses are popular ornamental plants all over the world. *Rosa damascena* Mill., also known as the damask rose, is a well-known scented rose species cultivated to produce essential oil. The essential oils obtained are high in volatile organic compounds (VOCs), which are in demand across the pharmaceutical, food, perfume, and cosmetic industries. Citronellol, nonadecane, heneicosane, caryophyllene, geraniol, nerol, linalool, and phenyl ethyl acetate are the most important components of the rose essential oil. Abiotic factors, including as environmental stress and stress generated by agricultural practises, frequently exert a selective impact on particular floral characteristics, hence influencing the overall quality and quantity of rose products. Additionally, it has been observed that the existence of stress exerts a notable impact on the chemical composition and abundance of aromatic compounds present in roses. Therefore, understanding the factors that affect the biosynthesis of VOCs, especially those representing the aroma and scent of rose, as a response to abiotic stress is important. This review provides comprehensive information on plant taxonomy, an overview of the volatolomics involving aromatic profiles, and describes the influence of abiotic stresses on the biosynthesis of the VOCs in damask rose.

## 1. Introduction

*Rosa damascena* Mill. (damask rose), in the Rosaceae family, is native to Europe and Middle Eastern nations, including Iran and Turkey [1,2]. It is one of the most well-known varieties of fragrant roses. Since it is rich in essential oils, it has an intense odour. The damask rose’s dried petals are used commercially for the production of rose oil, rose water, rose concrete, and rose absolute, as well as flavourings, cosmetics, and health products [3,4,5]. They are also cultivated as ornamental landscape plants [6,7]. Rose oil is one of the most expensive essential oils on the global market due to its low oil content, as one kilogramme of rose oil can be extracted from approximately 3000 kilogrammes of rose petals [8], and a shortage of natural and synthetic substitutes [9]. Approximately 4.5 tonnes of rose oil are produced annually on a global scale [10]. In the period from 2019 to 2025, the global rose oil market is anticipated to expand at a CAGR of 6.8%, reaching USD 442 million [11]. Rose oil has also been documented to have pharmacological properties, including antioxidant, antibacterial, and antimicrobial [12,13], anti-inflammatory [14], anticancer [15], and anti-HIV [16] properties. Essential oils contain predominantly alkanes, alcohols, phenols, terpenes, and terpenoids. Nonadecane, eicosane, heneicosane, heptadecane, and octadecane are the most abundant alkanes, while phenylethyl alcohol, citronellol, geraniol, neral, linalool, and farnesol are the most abundant terpene and terpenoid compounds [17,18]. Terpenes are the primary volatile organic compounds (VOCs) found in roses, and they play a significant role in determining the aroma or aromatic profile of roses. These compounds can be consistently released from several parts of the plant, including flowers, leaves, fruits, roots, and other minor organs, such as pollen [19]. Nevertheless, this aroma has the ability to elicit plant defence mechanisms, attract pollinators, and provide sensory pleasure for humans [20,21]. The olfactory characteristics of roses are distinguished by a pleasant and fragrant aroma, which exhibits possible nuances of fruity, spicy, earthy, and herbal elements. The olfactory characteristics may display variation between several cultivars of roses. In conclusion, the aromatic profile has played a significant role in the selection processes for its utilisation in various industries such as cosmetics, the perfume business, and medicine.

Nonetheless, biotic and abiotic stresses negatively impact plant growth, development, and agricultural productivity. According to current climate prediction models, plants are subject to stronger environmental stresses, such as salinity, drought, and mineral deficiency [22]. Furthermore, stress factors frequently exert opposing selective pressures on specific floral characteristics such as pigmentation, nectar content, and aroma [23]. Drought stress is one of the most serious stresses and it is known to harm horticultural produce, including floriculture. In addition to the evidence of reduced growth and yield losses, drought stress also impacts the biosynthesis of secondary metabolites [24,25]. They are necessary for the plant to interact with its environment for adaptation and defence against viruses, parasites, and herbivores, but do not play a fundamental role in the maintenance of life processes in plants [23,24]. Terpenes, phenolic acids, flavonoids, alkaloids, tannins, and VOCs represent the principal secondary metabolites commonly found in plants [26,27,28]. Nevertheless, a total of 1700 compounds have been recognised as VOCs, which play crucial roles in the environmental adaptability and survival of plants [29]. They are released directly from the blossoms to entice pollinators and promote environmental stress tolerance [30]. It is expected that, under such stressful circumstances, plants will produce VOCs at higher concentrations and for longer durations [31].

For damask rose farmers, knowledge of the factors influencing volatile metabolomics is crucial. Changes in these variables may contribute to the evolution of diverse plant genes that favour the adaptability to environmental conditions at crop locations, resulting in alterations to the quantity and quality of VOCs [18]. While the interest is overwhelming regarding the exploitation of the VOCs from roses and the concerns of global warming, there is not much information regarding the biosynthesis of VOCs, especially those representing aroma and scent of rose as a response to abiotic stress. This review therefore gives an overview of the volatolomics involving aromatic profile and describes the influence of abiotic stresses on the biosynthesis of the VOCs in damask rose. The potential benefit of this review is to disseminate a comprehensive understanding of the various elements that impact the biosynthesis of aromatic compounds. This knowledge holds significant relevance for those associated with rose farming as well as producers within the perfume, food, pharmaceutical, and cosmetic industries.

## 2. Taxonomy of Damask Rose

**Figure 1:**
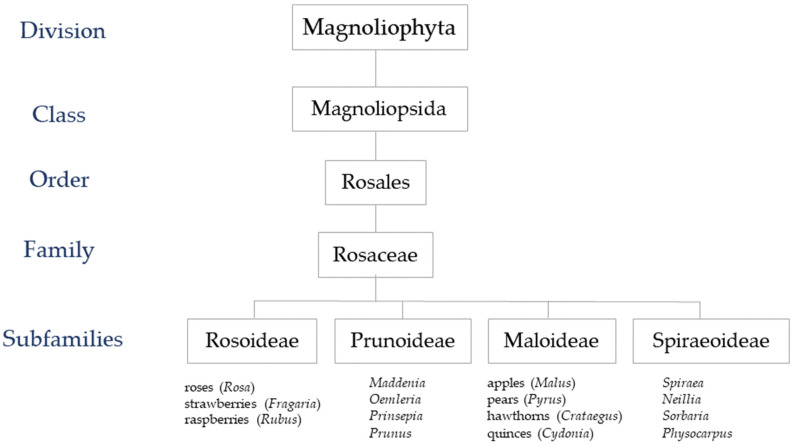
Scientific classification of roses.

Rosaceae is the 19th largest plant family with more than 3000 species from at least 100 genera [32]. The distribution of families is varied but concentrated, especially in the northern hemisphere. The most significant genera are *Rosa* (Rose), *Fragaria* (Strawberry), *Malus* (Apple), *Pyrus* (Pear), *Prunus* (Almond, Apricot, Cherry, Peach, Plum, and others), *Rubus* (Blackberry), and *Cydonia* (European quince) [33,34]. Within the same family, taxonomic classifications have varied significantly due to the numerous proposed categorisation approaches over the past century [35]. The traditional classification of Rosaceae includes four subfamilies: Rosoideae, Amygdaloideae (also known as Prunoideae), Maloideae, and Spiraeoideae (Figure 1) [36]. Rosoideae consists of both woody and herbaceous taxa with the well-known members including *Rosa*, *Fragaria*, and *Rubus*. Amygdalo ideae mostly comprise the *Prunus* genus, which includes peaches, plums, cherries, and almonds. *Malus*, *Pyrus*, *Crataegus*, and *Cydonia* are within the Maloideae. Lastly, the Spiraeoideae consists of woody and herbaceous plants and some well-known ornamental taxa (*Spiraea*) [37].

**Figure 2:**
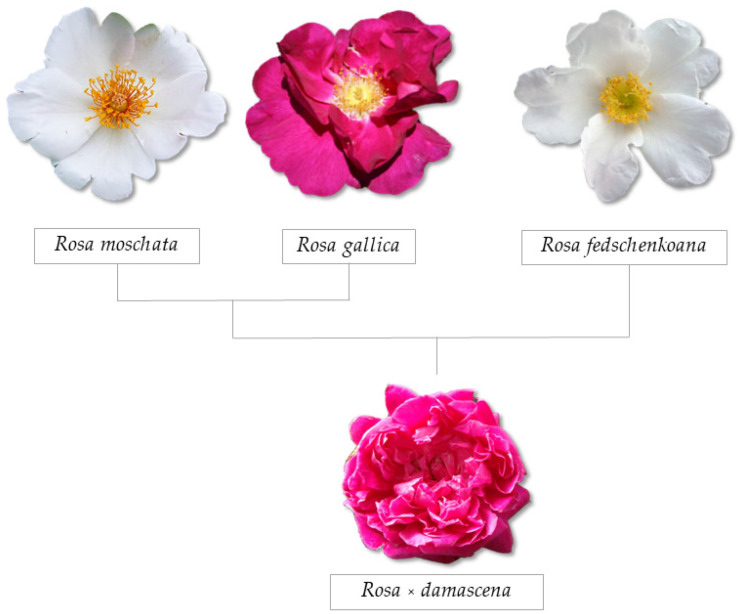
The pedigree of *Rosa damascene*.

In the genus *Rosa*, almost 200 species and more than 18,000 cultivars have been documented [38]. Within the genus, it is divided into four subgenera based on their fruit structure, three of which are monotypic: *Hulthemia* (Dumort.) Focke, *Hesperhodos* Cockerell, and *Platyrhodon* (Hurst) Rehder. The fourth subgenus, *Rosa*, harbours about 95% of all species and is subdivided into 10 cultivars, including *R. canina*, *R. chinensis*, *R. foetida*, *R. gallica*, *R. gigantea*, *R. moschata, R. multiflora*, *R. phoenicea*, *R. rugosa*, and *R. wichuraina*. The modern cultivars are mostly interspecific hybrids derived from these cultivars [39,40]. There are several hypotheses surrounding the ancestry of the damask rose. However, it is believed that the plant has a triparental origin, with *R. moschata* as the maternal ancestor and two successive crossings (*R. moschata* × *R. gallica*) × *R. fedschenkoana* Regel (Figure 2). The complex allotetraploid *R. damascena* used for cultivation has a steady chromosomal number (2*n* = 4*x* = 28) [41,42].

**Figure 3:**
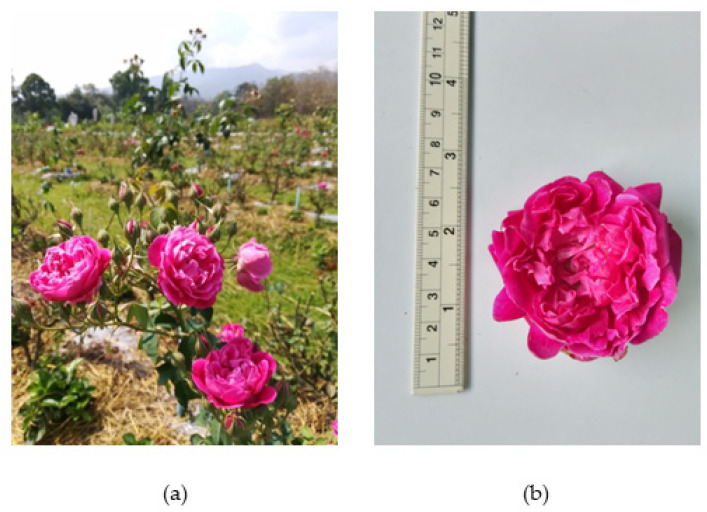
Morphological characteristics of damask rose: (**a**) whole plant; (**b**) flower.

Damask rose is one of the few fragrant species among the hundreds in the *Rosa* genus [40]. It is a shrub that may attain a height of 2.5 metres (Figure 3a). The stems are extensively covered with curved spikes, and a four-year-old or older plant can yield between 500 and 600 flowers annually. The flowers have approximately thirty petals that range in colour from pale to medium pink to pale crimson (Figure 3b) [43]. The optimum growing temperature is between 15 and 21 degrees Celsius [44]. Common asexual methods of propagation include suckering, hardwood and semi-hardwood cutting, sprouting, and grafting [45].

## 3. Biosynthesis of the Aromatic Volatiles in Damask Rose

The VOCs can be applied as fragrances, flavouring, and aromas in pharmaceuticals, foods, and biofuel feedstock. Under particular environmental conditions, plants rely on VOCs for survival and environmental adaptation, and flowers release VOCs directly to attract pollinators. When released from vegetative tissues in constitutive or induced conditions above or below ground, VOCs may repel pathogens and herbivores and/or attract their natural adversaries. In addition, VOCs are capable of inducing immunological responses in nearby plants of the same or different species, thereby affecting the entire regional plant community. In addition to their roles in biotic interactions, VOCs can also mediate resistance to abiotic stresses like cold and high temperatures [30]. Flowers come in different shapes, colours, and odours. Floral scents are complex mixtures of VOCs and low molecular weight lipophilic metabolites that are released into the atmosphere by a variety of mechanisms, such as diffusion through the cuticle and/or stomata, active (ATP-activated) transport through cell membranes by protein channels, or by the destruction of tissue structures [46,47]. Terpenoids, phenolics, and nitrogen-containing compounds are the primary contributors to the aroma released by flowers [30,48]. Terpenes are the isoprene (C5) molecules arranged in a head-and-tail configuration; typically they are produced in plants through the 2-methylerythritol 4-phosphate (MEP) pathway (producing monoterpenes (C10), diterpenes (C20), triterpenes (C30), and tetraterpenes (C40)) and the mevalonate (MVA) pathway (producing sesquiterpenes (C15)). The cytosol synthesises sesquiterpenes and triterpenes, while the plastids produce monoterpenes, diterpenes, and tetraterpenes. A further crucial category of secondary metabolites that contribute to floral aromas is phenolics. These aromatic compounds consist of hydroxyl groups that have been methylated or glycosylated. There are five identified subgroups of phenols, including phenolic acids, flavonoids, coumarins, lignins, and tannins. All phenolic chemical precursors are produced through the shikimic acid and malonic acid pathways. The shikimic acid pathway is used to produce most phenolic compounds in plants. Alkaloids, which contain heterocyclic nitrogen atoms, are the third-most-abundant form of secondary metabolites in plants. In addition to alkaloids, two significant families of N-containing secondary metabolites, cyanogenic glycosides and glu-cosinolates, are also present [48].

Damask rose is one of the most important aromatic species in the Rosaceae family, and its essential oils along with high-value products are utilised extensively in the medicinal, food, perfume, and cosmetic industries. The flowers are rich in vitamin C, and their essential oils have sedative, antiviral, and antibacterial properties [49]. Citronellol, non-adecane, heneicosane, -caryophyllene, geraniol, nerol, 1-nonadecane, E-citral (gerani-al), -pinene, linalool, and phenyl ethyl acetate are the most important components of the essential oil of damask rose (Table 1). These prominent scent compounds released from the flowers of damask rose serve as an important quality indicator and primarily determine the price of rose oil [4,50]. Nevertheless, the main components of musk rose (*Rosa moschata*) are phenylethyl alcohol, 1-nonadecene, heneicosane, and n-nonadecane. However, smaller quantities of phenylpropanoids and other chemicals can be detected [51].

## 4. Agronomic Aspects Influencing the Biosynthesis of Aromatic Compounds in Roses

During flowering, the damask rose requires moderate temperatures and humid air in order to produce high oil content. This rose has been widely cultivated in many countries, including Iran, United States, United Kingdom, Bulgaria, Turkey, Japan, and India. It is predominantly grown in warmer climates, typically at an altitude of 300–1500 m. [18,57]. Since ancient times, damask rose has been cultivated for various purposes, in Iran having a long history of producing and exporting essential oils around the world [18,58]. Although it is unclear why plants produce essential oils, these oils are generally the byproducts of major metabolic processes in plants, particularly under stress conditions [59,60]. Due to the evolution of various plant genes that promote adaptation to environmental conditions at crop sites, it is possible to observe an alteration in the quantity and quality of essential oils as a result of the environmental interactions of these plants [61,62,63]. The method of propagation, time of harvesting, air temperature, relative humidity, intensity of sunlight, flower stages, day period of harvesting, harvest procedures, time and level of pruning, storage of plant material, and method of distillation are the most influential agronomic factors on essential oil quality [43]. It was discovered that nutrient provision and irrigation are two of the most important factors influencing the production of essential oils by plants in a field. As is common knowledge, soil characteristics also impact plant growth and development, and the quantity and quality of yields [18]. The chemical elements in the rhizosphere (such as mobile phosphorus and potassium (K) content) are incorporated into the enzymes participating in the biochemical reactions occurring within plants. Soil chemistry can thus influence the essential oil composition (such as linalool, citronellol, geraniol, eugenol, and so on) as well as the distribution of chemotypes [64]. Water is also an important environmental factor that affects plant growth and yield quality, and the quantity of essential oils.

Crude distillation of roses for oil is thought to have originated in Persia in the late 7th century A.D., and spread to the provinces of the Ottoman Empire later in the 14th century [2]. As a result, a wide range of diversity is expected in Lebanese damask rose landraces. Different gene complexes favouring adaptation to the environmental conditions may have evolved over the period of time, leading to the diversity in damask roses and the complexity of their aromatic profiles [65]. The choice of organic production of an essential oil crop over conventional production is frequently reported expensive and tedious for agricultural producers, but the results are especially important for the production system, and to obtain sufficient quantities of good quality produce [66]. It has been found that the nutrient concentrations of damask roses from conventional gardens are significantly higher than those from organic gardens, particularly for nitrogen (N), K, calcium, and iron concentrations, representing the accumulation of inorganic substances. Paclobutrazol, an antigibberellic, combined with N supplied in appropriate amounts, and the micronutrients Mn^2+^, Zn^2+^, and Cu^2+^, improved flower bud formation and flowering, and rose oil yield having a higher percentage of citronellol [67]. Farmers must decide upon the choice of conventional or organic agricultural system based on the benefits and challenges of the agricultural sector. However, one of the major challenges in organic rose oil cultivation is low resistance to major diseases and pests [10]. The system and agricultural practices used in the cultivation can have a significant impact on the quality of cosmetic rose products and food supplements. The essential oil from organic farming provides higher linalool and geraniol content and lower β-citronellol + nerol concentrations than conventional farming. It has also been found that combining ecofriendly agricultural practices in organic private farms for damask rose cultivation resulted in higher antioxidant activity values in rose phytoconstituents from rose methanolic extracts compared to those from conventional agricultural systems [68].

## 5. Influence of Abiotic Stressors on Aromatic Profile of Damask Rose

Plants constantly face a variety of biotic and abiotic environmental stresses and thereby grow and survive in adverse environments using indirect and direct defensive mechanisms [69]. Although natural acclimation responses in flowering plants include changes in these traits, unusual changes in an environmental factor (e.g., temperature, herbivory) may alter floral biochemistry to the point where their ability to attract pollinators is compromised and reproductive fitness is lost. Lower levels of attractant metabolites and higher levels of warn-off substances are common trade-offs, disrupting the established balance acquired by specific floral phenotypes within their respective communities [47,70].

**Figure 4:**
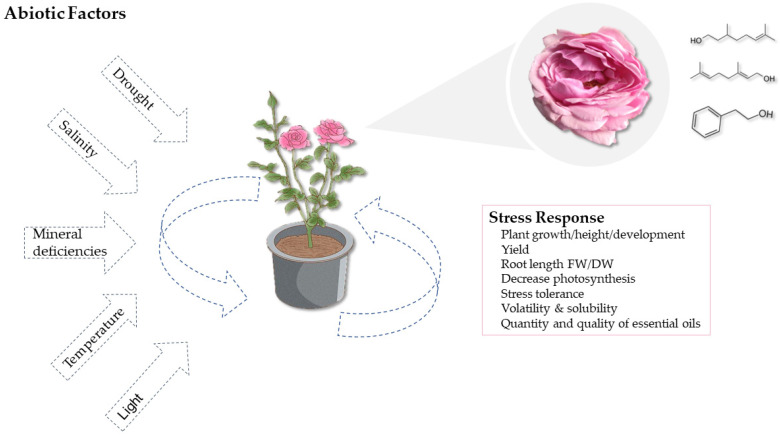
Abiotic factors influencing rose plant growth.

Abiotic stress and climate change are significant constraints to crop production. The science of plant abiotic stress tolerance encompasses all studies on nonbiotic environmental factors or stresses that can cause an impact on plant species [71]. Plants frequently face various abiotic stresses, such as temperature, dehydration, salinity, nutrition, UV radiation, and heavy metal toxicity. These stressors significantly impact the development of plants, including plant yield, morphology, and growth, and development is complex in today’s changing climatic scenario (Figure 4). Crop yield and quality limitations have accelerated with increased human birth rate, posing an additional threat to natural resources [72]. Abiotic stresses also limit global food demand, and a homeostatic environment promotes the discovery of underlying mechanisms for the development of climate-resilient crops [73]. Extreme temperature and light density, radiation (UV-B and UV-A), water stress (flooding, drought, and submergence), mechanical factors, chemical factors (heavy metals and pH), salinity due to excess Na^+^, excess or deficiency of essential nutrient elements, gaseous pollutants (sulphur dioxide, ozone), and other commonly occurring stressors in lesser amounts under field conditions are examples of abiotic stressors in horticultural production. Stresses such as heat and drought can have unique effects on plant-stress physiology that cannot be elucidated by a single type of stress factor [74]. As a result, a variety of physiological relationships can be projected, necessitating individual innovative approaches. A specific type of environment for one plant species may not be adaptable to another, and external abiotic or biotic factors can impose stress on plants, depending on their genetic makeup and adaptive response. The interaction of environment and abiotic stresses may plague plants, and these factors impose epigenetic influence on plant behaviour and progeny, bringing new insights to revisit plant–environment interactions. Volatile terpenoids released from flowers attract pollinators and protect against biotic and abiotic stresses [75]. For example, geraniol has antibiotic activity and can be detected with high sensitivity by honeybee antennae [76]. The β-ocimene and linalool are common pollinator attractants with antibacterial properties [77]. (E)-α-farnesene extracted from *Brassica rapa* flowers attracted bees rather than butterflies, and (E)-β-caryophyllene promotes plant fitness and aids in the defence against pathogenic bacteria [78,79]. Nonbiotic environmental pressures will continue to pose a significant challenge to sustainable agricultural practices and the natural environment. The current rate of rapid industrialisation and urbanisation, the growing human world population, coupled with deteriorating soil, air, and water resources, as well as climate change, global warming, and greenhouse gas effects, is negatively affecting global crop productivity. The major tasks ahead of us in plant-stress physiology and sustainable crop improvement are to compile the very foundation of systems-level information on plant abiotic stress response and signal transduction pathways, characterise abiotic stress defence networks, and to produce climate-resilient crop plants having more yield and quality attributes to feed the world’s growing population [80]. Environmental factors are responsible for enhancing the production of low floral and essential oil yields, which are a major problem for farmers and manufacturers alike. This article presents an extensive review of the VOCs in damask roses when exposed to various stressors.

### 5.1. Drought Stress

Drought stress is one of limitations in agricultural production because it is generally harmful to plant growth and must be studied in areas where water is scarce for agriculture and in areas that rely on rainwater [27]. Cell wall maintenance has been linked to drought resistance. In fact, a decrease in water potential can be avoided by shrinking cellular volume in water-limited environments due to the elasticity of plant cell walls, and plants have developed a variety of mechanisms to tolerate drought stress, including a modified root-to-shoot ratio, smaller and fewer leaves, and altered stomatal function. Drought reduces leaf area due to reduced cell expansion and cell division, leaf rolling, and death of apical leaf portions. Plants with larger leaf areas have a higher transpiration-to-evaporation ratio, resulting in more efficient water use. A variety of biochemical, physiological, and chemical changes were induced by drought stress, resulting in membrane injury and cell function loss, together with a decrease in plant growth [81]. Drought severity and frequency are expected to increase due to climate change-induced modifications in typical precipitation patterns. Water scarcity sends a chemical signal through xylem sap to the aerial system, causing partial stomatal closure to prevent water loss by evaporation. As a result, plants adopt a water-saving strategy that reduces intracellular CO_2_, lowering the amount of NADPH^+^, H^+^, and ATP available for CO_2_ fixation within the Calvin cycle, lowering NADP^+^ regeneration and influencing the photosynthetic electron transport chain [82]. Not only does the production of essential oil in plants rely on the metabolic mode of resource tissue, but it may also influence stress factors. It is necessary to use appropriate irrigation to produce each pharmaceutical plant to extract its essential oil and other bioactives. While the identification of commercial and drought-resistant species is critical, it is also necessary to investigate the ratio of available essential oil in pharmaceutical plants and observed water scarcity. Drought stress can stimulate a wide range of aromatic components, with the number of aromatic components stimulated by fresh leaves increasing in proportion to the severity of the drought [83]. However, in a study of drought stress on essential oil composition of damasks rose found no significant difference of chemical profile. In fact water stress increased yield of the essential oil [84]. Hassan, et al. [85] investigated the application of Spermine (Spm) or Spermidine (Spd) on some physiological and biochemical processes to comprehend the potential mechanisms concerning the alleviation of water stress in damask rose. They concluded that under water stress, foliar administrations of Spm or Spd at a concentration of 0.5 mM improved growth characteristics, relative water content (RWC), chlorophyll content, and stomatal conductance. In addition, the proline content and catalase (CAT) and superoxide dismutase (SOD) enzyme activities were enhanced by the application of Spm or Spd. As a result, the production of hydrogen peroxide (H_2_O_2_) was inhibited, as was the accumulation of malondialdehyde (MDA); consequently, membrane stability was maintained, and water stress damage was mitigated. Farahani, et al. [86] reported that applying Si to the leaves at a concentration of 0.2% in the spring and summer, especially when under water stress conditions, is a good way to increase the essential oil content and concentration of geraniol, citronellol, eugenol, and methyl eugenol, which are the main compounds in rose oil. Kiymaz, et al. [87] found that the response in yield and quality of the essential oil of *R. damascena* was a direct result of the relatively constant water use efficiency. According to the results of the study, irrigation water level at 0.50 and 80 kg ha^−1^ treatment produced the highest yield of fresh flowers per plant. As fertiliser level increased and irrigation level decreased, fresh flower yield, oil yield per plant, plant height, number of branches, and leaf area decreased. Few changes were observed in the quality of essential oils as water stress increased with less applied water. Yousefi [88] evaluated the flower yield and essential oil content in 49 different Iranian damask rose landraces. It was found that most landraces originating from temperate, warm temperate, and arid regions produced a greater flower yield and essential oil than those originating from cool temperate, semi-arid and humid regions. In summary, drought stress poses a significant threat to crop production, impacting damask rose growth and essential oil production. Understanding the mechanisms of drought resistance and implementing appropriate irrigation techniques are crucial for maintaining optimal plant performance and maximising rose oil yields in drought-prone regions.

### 5.2. Salt Stress

In general, little is known about the physiological nature of salinity impacts and the biochemical mechanisms occurring under salt stress. Salt stress involves the metabolic processes particularly in protein synthesis and inorganic N incorporation into amino acids, which is largely dependent on the type and amount of salt used and the plant species being studied [89]. Salt stress can occur as a catastrophic occurrence, be applied constantly or intermittently, and become progressively more severe at any point throughout development [90]. Rock erosion, capillary increase in brackish groundwater, water inlets from the sea by the coast, restricted soil drainage, little rainfall, high evaporation rates, and/or climate change along with fertilisation overuse are the main causes of primary salinity [91]. Whether salt shock or stress will occur depends on how the plants are exposed to salinity [92]. Both the osmotic and ionic parts of salt stress and salt shock generally stop plants from growing. Osmotic stress limits water intake, which results in turgor loss and a rise in ion concentration within the cells. Plants can become toxic from the ionic component, which can also induce cell death from an excessive ion build-up. Ionic changes occur when there is an imbalance in solutes, such as when the ratio of K^+^ to Na^+^ decreases and Na^+^ and Cl^−^ increase in the cytosol [93]. Roses are typically considered to be susceptible to salinity. However, the availability of excellent water is limited in many rose-growing regions, and soil salinisation is frequent. The effects of salinity on *Rosa* species depend on salinity type and concentration, cultivation method, substrate type, irrigation system, species or cultivar, and rootstock selection [93]. Most often, higher soil salt concentration adversely affects growth and flowering, which interferes with the appearance and aroma quality of several rose species [94]. Rose landscape cultivars such as *R. chinensis* do not blossom but instead go directly into hibernation under conditions of salt stress [95]. When mini roses (*R. hybrida* L. ‘Red Imp’) are exposed to salt stress, they bloom later and have fewer flowers per plant [96]. Additionally, when the saline level is moderate or high, garden roses like *R. hybrida* L. types (‘Caldwell Pin’, ‘Carefree Delight’, ‘Marie Pavie’, and ‘The Fairy’) produce fewer blooms [97]. Furthermore, salt stress impacts the water potential of plants. When *R. chinensis* was exposed to saline water, the amount of leaf water and dry matter decreased [98]. A rise in the salt content in irrigation water also hurts the height, stem diameter, and dry matter production of rose plants [99]. The effect of various NaCl concentrations on plant growth has been studied and the quality of essential oils from *R. damascena* var. *trigintipetala* Dieck showed that at a concentration of 500 ppm, it may be effective for enhancing and stimulating the quality of essential oil constituents such as citronellol, geraniol, and phenyl ethyl alcohol. The chemical alterations caused by salinity may represent an adaptation to this factor. In this instance, exposing rose plants to salinity stress may be a viable method for enhancing essential oil production. In addition, mitigation of salt-stress effects by foliar application of moringa leaf extract (MLE) or salicylic acid (SA) was found to enhance the growth, relative water content, proline content, total phenolic content, activity of antioxidant enzymes CAT and SOD, and chlorophyll content in *R. damascena* var. *trigintipetala* Dieck [100]. Omidi, et al. [101] found that the application of SA, even at low concentration (0.5 mM), could mitigate the detrimental effects of salinity stress in *R. damascena*. Salinity increased the activity of antioxidant enzymes CAT and SOD, as well as the concentrations of proline, protein, and glycine betaine. Overexpression of antioxidant genes (ascorbate peroxidase (APX), CAT, peroxidase (POD), Fe-SOD, and Cu-SOD) played a significant role in Damascus rose salt tolerance. Additionally, 0.5 mM SA increased the activity of enzymatic and nonenzymatic systems alongside salinity tolerance. Attia, et al. [102] found that *R. damascena* salt tolerance at 100 mM NaCl was correlated with the maintenance of high water and chlorophyll contents. Salt impeded lipid peroxidation by elevating MDA and H_2_O_2_ levels. Leaf SOD, CAT, and guaiacol peroxidase activities decreased in response to varying concentrations of NaCl, concurrent with a decrease in polyphenol, tannin, and flavonoid content.

### 5.3. Nutrient Stress

Nutrient management that incorporates organic and inorganic fertilisation has a positive impact on soil organic matter and available plant nutrients, resulting in long-term crop production. Furthermore, nutrient availability is important in this regard [103,104]. Producers are unable to maximise damask rose production and quality due to a lack of information and technical skill on the optimum levels of various nutrients. Macro and micronutrient treatments are critical in this regard [105]. Nutrient management is critical for economic and environmental sustainability. N is essential in the synthesis of plant constituents via the action of various enzymes. N fertilisation has an impact on both the quantity and quality of essential oil. In general, N applications increase oil yield in aromatic plants by increasing biomass yield per unit land area, leaf area development, and photosynthetic rate [106,107]. Phosphorus (P) is essential for many metabolic processes. It is found in nucleic acids, phospholipids, coenzymes, Deoxyribonucleic acid (DNA), nicotinamide adenine dinucleotide phosphate (NADP), and, most notably, adenosine triphosphate (ATP). It activates the coenzymes to produce amino acids, which are used in protein synthesis, and decomposes carbohydrate production in photosynthesis along with the glycolysis, respiration, and fatty acid synthesis. K is one of the most important elements for plant nutrition, accounting for 1–5% of crop dry matter; it plays a critical role in crop growth, yield, and quality. Plant height, main and secondary branch numbers, as well as leaf and stem dry weights increased with K and/or zinc (Zn) application, compared to control plants, with the combined treatments showing the greatest improvement [108]. Ghavam [18] reported the results of a study of the effects of irrigation water and soil physico-chemical characteristics on the essential oil yield of the damask rose. It was found that essential oils obtained from the Yazdel site had the highest concentrations of citronellol and geraniol (29.05% and 6.85%), which directly correlated with soil K and phosphorus content, and inversely correlated with soil acidity, electrical conductivity (EC) values, lime, N, and organic carbon contents. Additionally, micronutrients are important for the development of high yielding, high-quality products. However, only small quantities of micronutrients are required. Plants’ physiological and metabolic processes can be impacted by even small micronutrient deficiency [105,109]. Including Zn promotes photosynthetic and other metabolic activities that increase the levels of various plant metabolites needed for cell division and elongation [110]. Furthermore, the positive effects of nutrients, particularly Zn, on plant growth may be due to their requirement in tryptophan synthesis (as the precursor of Indole-3-acetic acid: IAA) and stimulation of IAA enzyme synthesis, as well as its effect on improving growth hormone biosynthesis [106].

Nutrient management is essential for balancing high floral yield with high quality oil production—two essential ingredients for profitable production of damask roses. It is a demanding crop that requires a steady supply of plant nutrients [108]. According to Shohayeb, et al. [111], *R. damascena* cultivated in the Shafa and Hada Mountains exhibited varied soil macro- and micronutrient concentrations, indicating that the soil water in each environment was alkaline. As a consequence, the rose produces citronellol, geraniol, and eugenol, three of the five components that make up high-quality rose essential oil. In another study, Kumar, Sharma, Kaundal, Sharma and Thakur [109] reported that foliar application of essential oils of damask rose using MgSO_4_, CuSO_4_, and ZnSO_4_ showed that MgSO_4_ + ZnSO_4_ at 1% resulted in higher citronellol and nerol content. In addition, the essential oils produced from the flowers of plants treated with ZnSO_4_ at 1% contain Z-rose oxide (2.3%), E-geraniol (26.5%), noadecene (1.5%), nonadecane (6.8%), docosane (0.6%), and heneicosane (2.6%) compared to other nutrient groups. In a similar study, Pal et al. (2016) investigated the interaction effects of nutrients and plant hormones on the secondary metabolite production of the damask rose. It was shown that the application of Ca(NO_3_)_2_ at 4 g L^−1^ increased the percentage of essential aromatic compounds, i.e., β-citronellol + nerol, linalool, E-geraniol, and Z-citral, thereby recommending the use of Ca(NO_3_)_2_ in combination with plant hormones to effectively improve flower yield and essential oil content in *R. damascena* [112]. Nevertheless, for plants to thrive, nutritional input is still required. The ecology or soil environment is essentially constrained. To increase yields and superior quality, it is vital to research and comprehend each region.

## 6. Molecular Bases of Abiotic Stress Response in Roses

**Figure 5:**
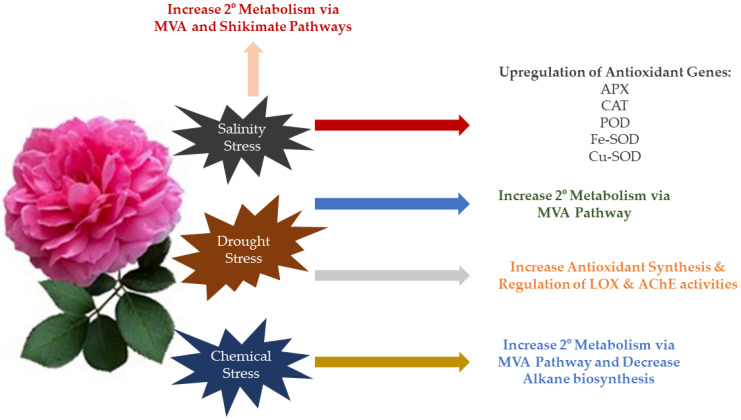
Molecular bases of *R. damascena* response to different abiotic stresses.

The molecular intricacies underlying the impact of combined abiotic stress on plant growth has been extensively studied. With the advent of increased bioinformatics research, there are now open-source datasets compiling the commonly expressed plant genes in response to abiotic stresses, such as salinity, drought, and metal exposure. Rose, being one of the important ornamental and cosmetic crops, has been reported for its limited yield and quality owing to drought. In *R. chinensis*, production of a cascade of osmotic protective agents such as sugars, amino acids, starches, and lipids were observed in response to drought stress. Genes linked to carbohydrate synthesis were found to be upregulated, which was evident with an increased accumulation of sugars in the plants exposed to drought. This was primarily reported to occur via the trehalose phosphate synthase (TPP1) pathway [113]. In *R. chinensis*, transcription factors belonging to the family AP2/ERF are believed to be responsible for rose’s drought stress regulation, while reports suggest the active involvement of a rose gene encoding the ethylene-responsive factor 109 (ERF109). However, there is a need for a detailed investigation into the molecular basis for its involvement. Furthermore, Jia, et al. [114] suggested that the basic helix–loop–helix (bHLH) transcription factors such as bHLH162 and bHLH35, and those from the MYB family, are particularly essential for the drought and cold responses in *R. chinensis.* In *R. damascena*, upregulation of antioxidant genes such as the APX, CAT, POD, Fe-SOD, and Cu-SOD was found to occur in response to salinity tolerance [101]. Hessini et al. [82] reported that the drought tolerance in *R. damascena* was mediated mainly via the increasing synthesis of antioxidants coupled with an orderly regulation of lipoxygenase (LOX) and acetylcholinesterase (AChE) activities. In another study, El-Sharnouby, et al. [115], concluded that the exposure to salinity stress stimulated and improved the quality of damask rose essential components such as citronellol, geraniol, and phenylethyl alcohol, via the MVA and shikimate pathways, respectively. In a similar study, foliar-applied silicon (Si) and water deficit stresses were found to influence the floral essential oil composition of *R. damascena.* Based on numerous reports, there is a suggestion that the utilisation of the is application results in an increase in methyl eugenol and n-heptadecane, along with a decrease in alkane compounds. Additionally, drought stress leads to a 1.5-fold increase in geraniol, eugenol, and citranolol, implying the effect of abiotic stress on the aromatic profile of the crop [116]. Therefore, there is a need for extensive evaluation of the mechanistic bases of abiotic stress response by damask roses to understand their functioning and improve yield and quality. Figure 5 illustrates the overall molecular response of damask rose to abiotic stressors.

## 7. Conclusions

In conclusion, abiotic stresses influence the aroma of damask rose. This review highlights the abiotic stress conditions of drought, salinity, and nutrient deficiency. Drought stress can stimulate a wide range of aromatic components, with the number of aromatic components increasing. The occurrence of drought conditions is an appropriate opportunity to enhance the essential oil content and concentration of geraniol, citronellol, eugenol, and methyl eugenol, which serve as the main compounds in rose oil. Exposing rose plants to salinity stress may be a viable method for enhancing essential oil production by improving and stimulating the quality of constituents such as citronellol, geraniol, and phenylethyl alcohol. Moreover, citronellol and geraniol concentrations correlated directly with soil K and phosphorus content. We considered the main mechanisms involved in the most severe agricultural stress in crop production. The knowledge gained from this work collectively improves and increases our understanding of stress factors affecting the biosynthesis of the VOCs in damask rose. Finally, the above findings can be integrated with the already available information to improve plans for preventing stress(es) and improvising crop production impacted by the changing environment.

## Figures and Tables

**Table 1:** Chemical compositions and odour attributes of damask and musk roses.

Compounds	Odour Type	RI	% Peak Area		References
			Damask Rose	Musk Rose	
Monoterpene hydrocarbons					
α-Pinene	herbal, green	932	14.153 ±1.028	0.563 ± 0.155	[29,52,53]
β-Pinene	herbal, green	974	0.916 ± 0.710	0.036 ± 0.062	[29,52,53]
β-Myrcene	herbal, green	988	0.833 ± 0.434	-	[29,52,53]
α-Terpinene	herbal, green	1014	0.113 ± 0.070	-	[29,53]
γ-terpinene	herbal, green	1055	0.223 ± 0.152	0.106 ± 0.184	[29,53]
Limonene	herbal, green	1027	0.190 ± 0.113	0.123 ± 0.131	[29,50,52,53]
Terpinolene	herbal, green	1086	0.060 ± 0.000	-	[29,53]
Oxygenated monoterpenes					
Hexanol	fruity, floral	861	0.050 ± 0.000	-	[29,53]
Linalool	fruity, floral	1097	0.090 ± 0.014	-	[29,50,52,53]
dihydro-β-Ionone	woody	1435	-	0.181 ± 0.314	[29,53,54]
(E)-β-Ionone	floral	1482	-	1.431 ± 0.252	[29,53,54]
Terpinen-4-ol	woody, earthy	1174	0.070 ± 0.000	-	[53,54]
β-Citronellol	floral, rose	1226	35.530 ± 1.821	-	[29,50,52,53,54]
Neral	sweet, citrus	1238	0.615 ± 0.304	-	[52,53,54]
Eugenol	spicy	1353	-	1.151 ± 0.088	[52,53,54,55]
Geranial	fruity, floral	1268	0.345 ± 0.403	-	[29,52,53]
2-Phenyl ethyl acetate	floral	1254	-	0.377 ± 0.342	[53,54]
2-Phenyl propyl butanoate	fruity	1484	-	0.105 ± 0.182	[53,54]
Geranyl acetate	fruity, floral	1317	4.906 ± 0.833	-	[29,53]
Geranyl propanoate	floral	1496	0.640 ± 0.000	-	[53,54]
Sesquiterpenes hydrocarbons					
Sabinene	woody	970	0.340 ± 0.155	-	[53,54]
(E)-β-Farnesene	woody	1459	0.640 ± 0.000	-	[53,54]
α-Selinene	n/d	1498	1.580 ± 0.675	-	[53,54]
Hexadecen-1-ol	n/d	1866	0.180 ± 0.000	-	[52,53,54]
1-Tricosene	n/d	2285	-	1.972 ± 0.416	[53,54]
Oxygenated sesquiterpenes					
trans-Rose oxide	floral	1125	0.200 ± 0.096	-	[52,53,56]
Benzaldehyde	fruity	957	0.146 ± 0.081	-	[53,54]
p-Cymene	terpenic	1022	-	0.060 ± 0.104	[53,54]
Benzyl alcohol	floral	1029	0.186 ± 0.075	-	[53,54]
Benzene acetaldehyde	green	1041	-	0.103 ± 0.091	[53,54]
2-Phenylethyl alcohol	floral, rose	1110	36.600 ± 2.052	54.152 ± 1.340	[52,53,55]
Methyl eugenol	spicy	1402	0.185 ± 0.077	-	[52,53,54]
n-Octadecanol	n/d	2072	-	0.491 ± 0.450	[52,53]
Aliphatic hydrocarbons					
Hexacosane	n/d	2554	0.210 ± 0.000	-	[53]
1-Nonadecene	n/d	1874	-	15.576 ± 1.708	[29,50,53]
n-Nonadecane	n/d	1900	2.350 ± 0.385	8.147 ± 0.143	[29,50,52,53]
Heneicosane	n/d	2105	0.210 ± 0.000	8.175 ± 0.801	[50,52,53]
Tricosane	n/d	2285	-	1.196 ± 0.071	[50,53]
n-Tetradecane	n/d	1401	0.290 ± 0.000	-	[53]
n-Pentadecane	n/d	1499	-	0.140 ± 0.242	[50,52,53]
1-Heptadecene	n/d	1669	-	1.261 ± 0.152	[50,52,53]
n-Heptadecane	n/d	1698	0.646 ± 0.61	1.711 ± 0.067	[50,52,53]
Total			97.057 ± 0.347	99.556 ± 0.561	

RI: retention indices, n/d = no data. Peak areas (%) are the average mean of the reference data.

## Data Availability

The data that support the findings of this study are available from the author, S.R.S., upon reasonable request.
